# Veillonella Intrapulmonary Abscess With Empyema

**DOI:** 10.7759/cureus.45210

**Published:** 2023-09-14

**Authors:** Kayla Martinez, Gurvir Kaur Mangat, Noorulann Sherwani, Mark Glover DO, Marc Silver MD

**Affiliations:** 1 Surgery, Ross University School of Medicine, Pontiac, USA; 2 Internal Medicine, Ross University School of Medicine, Pontiac, USA; 3 Pediatrics, Ross University School of Medicine, Pontiac, USA; 4 Surgery, Trinity Health Oakland Hospital, Pontiac, USA; 5 Cardiothoracic Surgery, Trinity Health Oakland Hospital, Pontiac, USA

**Keywords:** lung abscess, chest tube, cardio thoracic surgery, video assisted thoracoscopic surgery, pulmonary empyema

## Abstract

A lung abscess is characterized as a clinical ailment arising from the localized suppurative necrosis of lung parenchyma. This condition primarily results from the complications of aspiration pneumonia due to anaerobic microorganisms originating from the oral cavity. Clinically, patients typically manifest symptoms such as fever, malaise, and a productive cough persisting over several weeks. The majority of lung abscess cases acquired within the community stem from anaerobic bacterial infections, often exhibiting a polymicrobial nature. We present a 51-year-old female with intrapulmonary abscess and empyema, with isolation of *Veillonella *species. She has a 25-pack-year smoking history. Two weeks prior to arrival at our facility, she experienced intermittent shortness of breath, fever, and subjective fever. Her primary care physician ordered an outpatient computed tomography (CT) which showed evidence of a large right-sided fluid collection. Initial chest X-ray at our facility revealed extensive opacification of the middle and right lower hemithorax, believed to be a large-sized pleural effusion with adjacent pneumonia or atelectasis. She was given a working diagnosis of right-sided empyema. Cardiothoracic surgery was consulted and video-assisted thoracoscopic surgery (VATS) was performed. A very large collection of grossly purulent material was evacuated and revealed a large intrapulmonary abscess. Over 400 cc of frank pus was collected and sent for microbiological analysis. Anaerobic culture demonstrated 3+ *Peptostreptococcus *species and 3+ *Veillonella *species. The genus *Veillonella *consists of a small, strictly anaerobic, gram-negative cocci that lacks flagella, spores, and capsules. This genus obtains energy from the utilization of short-chain organic acids that are present in the oral cavity and intestinal tract. Oral *Veillonella *is strongly associated with biofilms, causing human oral infectious diseases such as periodontitis and dental caries. Literature states that this organism has been isolated in a limited number of chronic pneumonitis cases. To date, the most common organism isolated from lung abscesses is *Streptococcus *in adult patients and *Staphylococcus aureus* in pediatric patients. We strive to elucidate the distinctive clinical presentation evident in this case, alongside a comprehensive understanding of the unusual pathogens identified in the disease's pathogenesis.

## Introduction

A lung abscess is characterized as a clinical ailment arising from the localized suppurative necrosis of lung parenchyma. This condition primarily results from the complications of aspiration pneumonia, due to anaerobic microorganisms originating from the oral cavity. While the exact incidence of lung abscesses remains undetermined, advancements in antimicrobial therapies have led to a significant reduction in mortality rates throughout the past century. Clinically, patients typically manifest symptoms such as fever, malaise, and a productive cough persisting over several weeks. The majority of lung abscess cases acquired within the community stem from anaerobic bacterial infections, often exhibiting a polymicrobial nature. We present a 51-year-old female with intrapulmonary abscess and empyema, with isolation of *Veillonella *species.

## Case presentation

**Figure 1:**
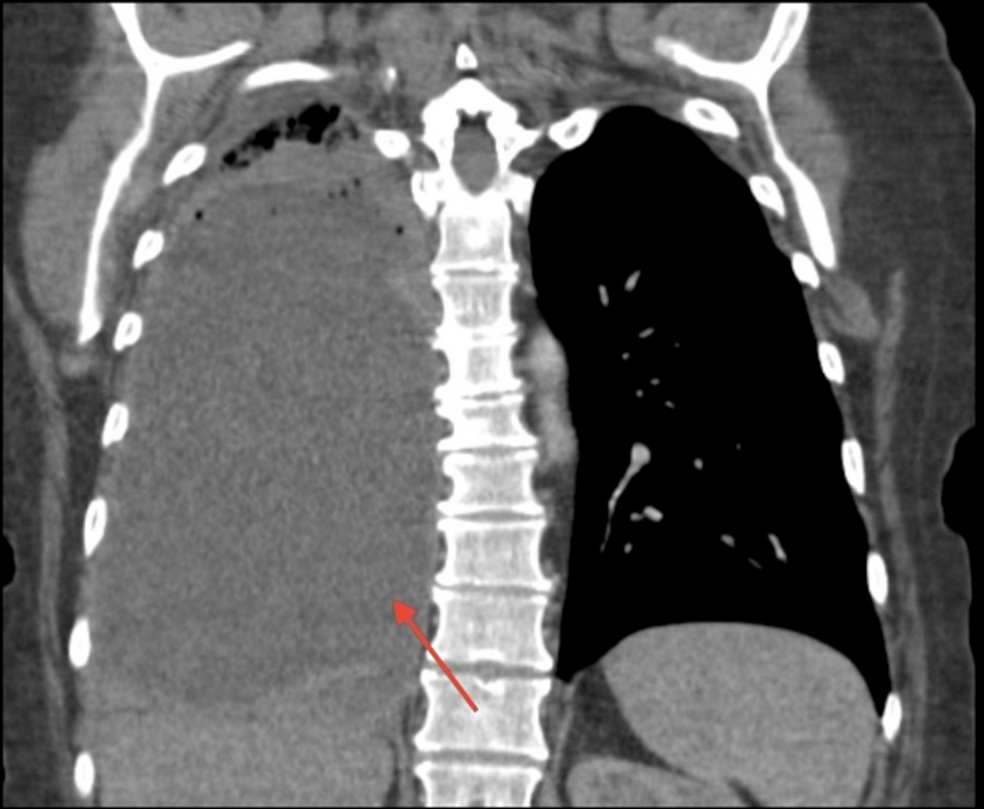
Outpatient CT of the Chest Red arrow indicating large right-sided large fluid collection. CT: computed tomography

This is a 51-year-old female with no significant past medical history. She has a 25-pack-year smoking history. Three months ago, she reported having right-sided musculoskeletal chest pain after lifting a heavy object while cleaning at home. At this time, she went to urgent care and was diagnosed with a muscle sprain. She was prescribed oral steroids and muscle relaxants, which provided her with minimal pain relief. Two weeks prior to arrival at our facility, she experienced intermittent shortness of breath, fever, and subjective fever. Her primary care physician ordered an outpatient computed tomography (CT) which showed evidence of a large right-sided fluid collection (Figure 1). She was advised to go to the emergency department for further evaluation.

**Figure 2:**
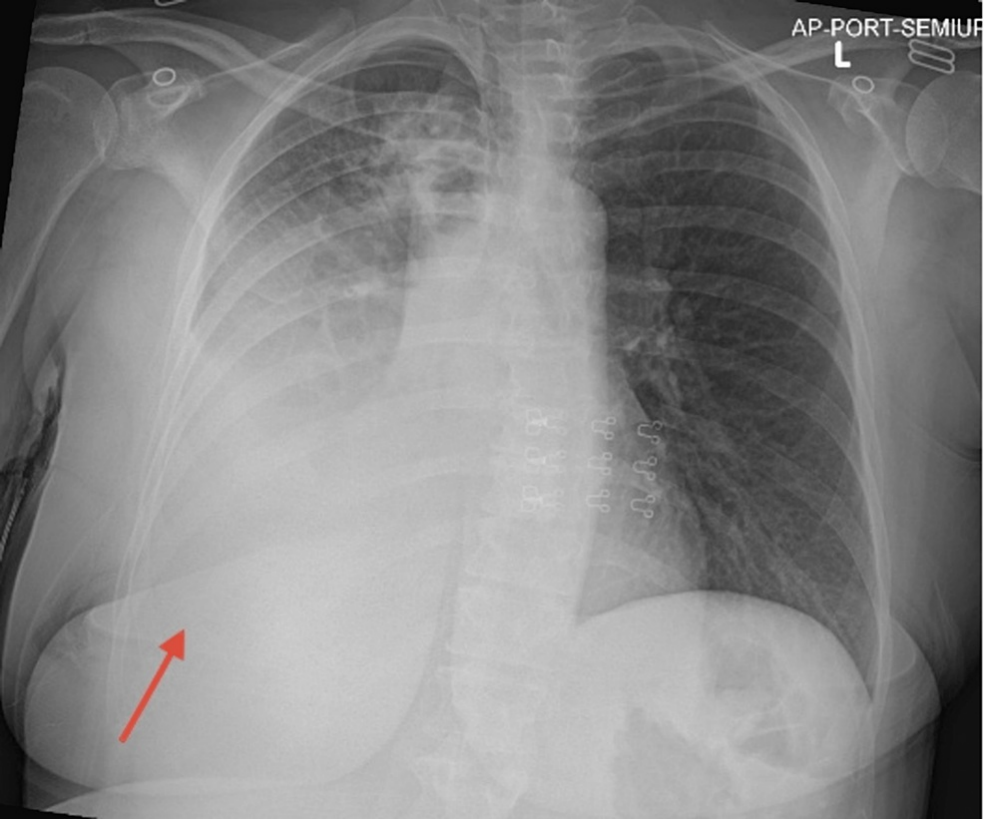
Initial Chest X-ray Upon Admission Red arrow indicating moderate to large right pleural effusion.

Upon arrival, her vitals were stable; BP: 107/72 mmHg, pulse: 112 bpm, respiratory rate: 18, temperature: 98.2 F, SpO2: 97% on room air. Physical examination was normal except for mild tachycardia at 112 bpm and absent right-sided breath sound and 2+ pitting edema up to the knees bilaterally. WBC was 16.5 K/mcL and Hb was 7.6 g/dL; Table 1 demonstrates the patient's abnormal lab values upon admission to our facility. Initial chest X-ray, as seen in Figure 2, demonstrated a moderate to large right pleural effusion with adjacent airspace opacities, opacifying the middle to right lower lung. The left lung was clear. No pneumothorax or enlarged cardiomediastinal contours were noted. She was given a working diagnosis of right-sided empyema. Infectious disease was consulted and intravenous (IV) vancomycin and piperacillin-tazobactam were given empirically.

**Table 1:** Patient’s Abnormal Lab Values Upon Admission All other labs were normal. WBC: white blood cells, RBC: red blood cells, RDW: red cell distribution width, NRBC: nucleated red blood cells

Result	Normal	Value
Sodium	135-144 mmol/L	133 mmol/L
Chloride	98-107 mmol/L	97 mmol/L
Creatinine	0.60-1.20 mg/dL	0.37 mg/dL
Calcium	8.6-10.3 mg/dL	8.3 mg/dL
WBC	3.7-11.0 K/mcL	16.5 K/mcL
RBC	3.80-5.20 M/mcL	2.64 M/mcL
Hemoglobin	12-16 g/dL	7.9 g/dL
Hematocrit	35-46%	25.2%
RDW	12-15%	17.2%
Platelets	150-450 K/mcL	735 K/mcL
Neutrophils Absolute	1.50-10.0 K/mcL	12.23 K/mcL
Immature Granulocytes Absolute	0.00-0.30 K/mcL	0.32 K/mcL
Monocytes Absolute	0.00-1.00 K/mcL	1.02 K/mcL
NRBC	<=0.2%	0.3%
NRBC Absolute	<0.01K/mcL	0.05 K/mcL

Cardiothoracic surgery was consulted and video-assisted thoracoscopic surgery (VATS) for evacuation of right-sided empyema was performed. During this procedure, a very large collection of grossly purulent material was evacuated and revealed a large intrapulmonary abscess. Over 400 cc of frank pus was collected and sent for microbiological testing and analysis. Two Blake drains were placed posteriorly, along with a 128 French chest tube placed anteriorly. Tissue was collected from the right pleural cavity. Anaerobic culture demonstrated 3+ *Peptostreptococcus *species and 3+ *Veillonella *species.

**Figure 3:**
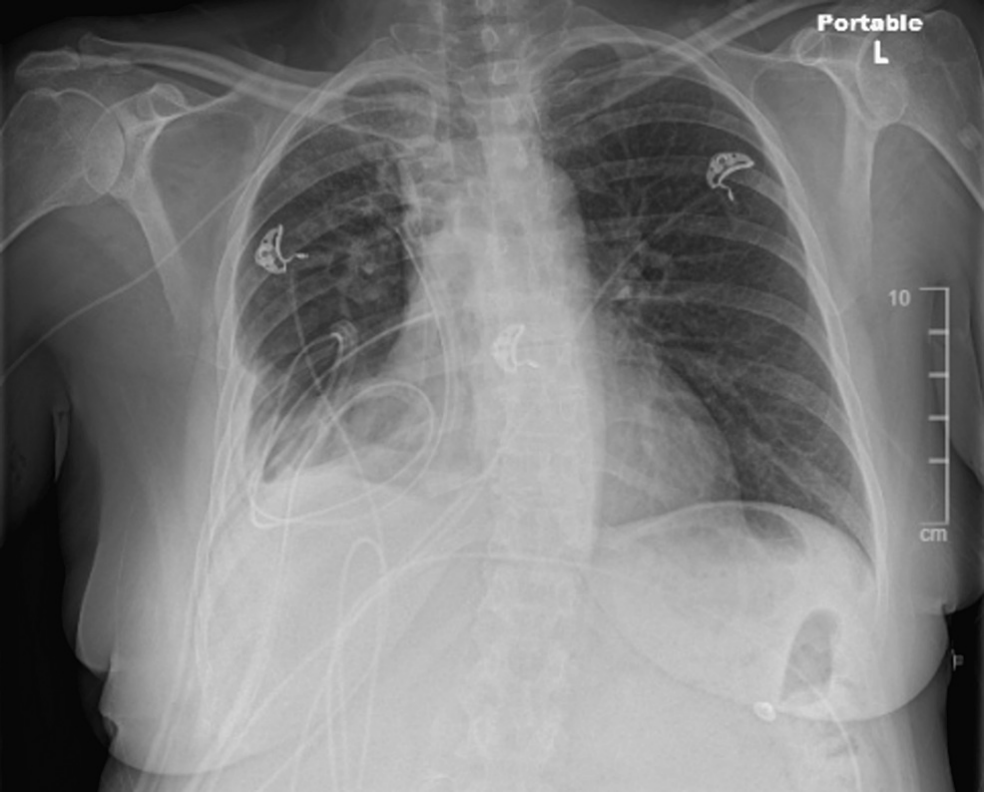
Chest X-ray Status Post-VATS, Postoperative Day 6 VATS: video-assisted thoracoscopic surgery

On postoperative day 1, she became hypotensive with a BP of 91/44 mmHg and bradycardic at 47 bpm. At this time, her WBC increased to 29 K/mcL. She was diagnosed with septic shock and given neosynephrine IV, which was later changed to Levophed. Her hypotension and heart rate stabilized shortly after. One unit of packed red blood cells was given due to hemoglobin decreasing to 6.9 g/dL status post-VATS. In Figure 3, her chest X-ray status post-VATS postoperative day six showed a right upper extremity peripherally inserted central catheter (PICC) line that terminates at the superior right cavoatrial junction. Two basilar thoracostomy tubes are present. A stable right basilar consolidation with a small pleural effusion was noted, and no evidence of pneumothorax was appreciated. The left lung is clear. Despite this event, our patient’s chest X-ray status post-VATS showed insignificant improvement from her initial imaging.

**Figure 4:**
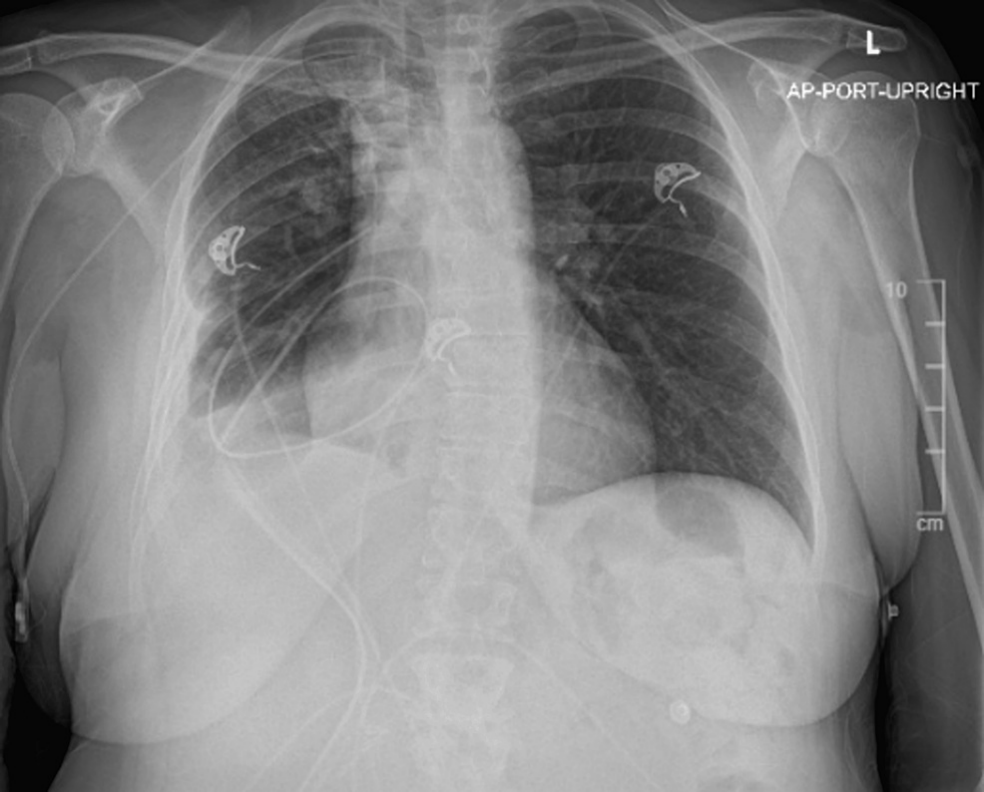
Final Chest X-ray Before Discharge

During her hospital admission, our patient was followed very closely by infectious disease. Upon admission to our facility, she was given piperacillin-tazobactam and vancomycin IV. On admission day, piperacillin-tazobactam was changed to cefepime IV. Upon receiving results for cultures, she was ultimately placed on ampicillin-sulbactam and ertapenem IV. Her final chest X-ray prior to discharge (Figure 4) from our facility showed a small, stable right pleural effusion with the unchanged position of the right-sided chest tube. Unchanged airspace disease was present in the right lower lobe, likely due to a healing infection. Right-sided PICC line with a tip projecting over the superior vena cava was noted. She responded well to this regimen and her chest X-ray demonstrated this prior to discharge. Admission to our facility concluded on day 12. Upon discharge, she refused placement to a subacute rehab, she wished to go home. She was discharged home on ertapenem via PICC line to continue for one month. She was given close follow-up for chest tube management with cardiothoracic surgery, pulmonary, and infectious disease.

## Discussion

Pulmonary abscess is defined as a local suppurative process that involves lung tissue necrosis. Lung abscesses can be classified as acute (less than six weeks) or chronic (more than six weeks). Lung abscesses are often unilateral, single and involve posterior segments of the upper lobes and the apical segments of the lower lobes as these areas are gravity-dependent when lying down [1]. Sinobronchial infections, dental procedures, oropharyngeal surgical procedures, and bronchiectasis have been identified as possible risk factors for lung abscesses (see Table 2).

**Table 2:** Common Etiologies, Pathogenesis, and Morphology of Lung Abscesses Ref: Kumar V et al. [2].

Etiology	Pathogenesis	Morphology	Additional Comments
Aspiration	Suppressed cough reflex, poor dental hygiene, protracted vomiting, severe dysphagia	Usually located in the right main bronchus	Pneumonia occurs first, then progresses to abscess formation
Primary Lung Infection	Post-pneumonic and transplant patients	Multiple, diffusely scattered, and located basally in the lungs	*S. aureus*, *Klebsiella pneumoniae,* *Pneumococcus*
Septic Embolism	Infected emboli arising from thrombophlebitis within the systemic venous circulation, vegetations from infective bacterial endocarditis	Multiple, diffusely scattered, and located in any region of the lung	Vegetations from right side of the heart lodge in the lungs
Neoplastic	Secondary infection in the bronchopulmonary segments due to obstruction from a primary or secondary malignancy	Squamous cell carcinoma of the lung is associated with cavitation formation and centrally located, whereas are lung adenocarcinoma is located peripherally	Histological tissue examinations are used to determine and confirm the type of primary lung malignancy
Other	Traumatic penetrations of the lung parenchyma Hematogenous seeding by pyogenic organisms	Varies	Direct extension of infections from the esophagus, spine, pleural cavity, or subphrenic space

The most common pathogens isolated from lung abscesses include both aerobic and anaerobic *Streptococci*, *S. aureus*, and several gram-negative organisms. Anaerobic organisms that are commonly found in the oral cavity, such as *Bacteroides*, *Fusobacterium*, and *Peptococcus *genera are isolated in over 60% of cases, however, over 90% of lung abscesses are polymicrobial. Aspiration of infective material has been identified as the most frequent cause of lung abscess formation. Risk factors that can increase a patient’s risk of aspiration include suppressed cough reflex related to acute alcohol intoxication, opioid abuse disorder, coma, anesthesia, and seizure disorder. Neurological deficits causing severe dysphagia, esophageal disease, protracted vomiting, and poor dental hygiene have also been identified. Aspiration first causes pneumonia, then progresses to tissue necrosis, cultivating the formation of an abscess in the lung parenchyma [2].

Lung abscesses are associated with a cardinal histological change of suppurative destruction of the lung parenchyma within the center of the cavitation. The abscess is usually filled with debris, however, if there is communication with an air passage an abscess can become partially drained resulting in an air-filled cavitation. Within necrotic debris, a superimposed saprophytic infection can result. Undiagnosed or continued infections lead to large, poorly demarcated, green-black, multilocular cavities that can progress to gangrene of the lung. In chronic lung parenchymal abscesses, extensive fibroblastic proliferation produces a fibrous wall. Abscesses can vary in diameter from millimeters to considerable cavitations of up to 6 cm. Pulmonary abscesses from aspiration are focal and occur in the right lung, due to the vertical nature of the right main bronchus. Abscess formation from pneumonia or bronchiectasis is usually multiple, diffusely scattered, and basally located in the lungs [2]. Primary lung abscesses result from direct infection of the lung parenchyma in an otherwise healthy patient who aspirates oral or gastric contents. Secondary lung abscesses occur from predisposing conditions such as bronchial obstruction from malignancy, thoracic surgery, or hematogenous spread [3].

The genus *Veillonella *consists of a small, strictly anaerobic, gram-negative cocci that lacks flagella, spores, and capsules. This genus obtains energy from the utilization of short-chain organic acids that are present in the oral cavity and intestinal tract. This genus has been divided into 13 species. *Veillonella atypica*, *V. denticariosi, Veillonella dispar, Veillonella parvula, Veillonella rogase*, and *Veillonella tobetsuensis *have been isolated from the oral cavity, specifically on the human tongue, buccal mucosa, and saliva. Oral *Veillonella *is strongly associated with biofilms, causing human oral infectious diseases such as periodontitis and dental caries. Shah et al. state that this organism has been isolated in a limited number of chronic pneumonitis cases [4]. Nour et al. reported one case of pulmonary septic emboli progressing to multiple focal lung abscesses successfully identified this organism [5]. To date, the most common organism isolated from lung abscesses is *Streptococcus *in adult patients and *S. aureus* in pediatric patients [5,6].

To diagnose intrapulmonary abscesses, plain radiographs and CT are most frequently used. In plain films, a lung abscess appears as a cavity containing a gas-fluid level. Abscesses appear round in shape in both frontal and lateral views. All margins of the abscess are equally seen, however, adjacent coexisting pathologies such as consolidation may distort this assessment. CT is regarded as the most sensitive and specific imaging technique to diagnose pulmonary abscesses. Administration of contrast allows for the identification of the abscess margins. Round shape and gas-fluid level can also be seen on CT. The walls of the abscess will appear thick with an irregular luminal surface. CT can also show additional structural involvement, more specifically, bronchial vessels and bronchi can be traced up to the abscess. To distinguish between pulmonary abscess and empyema CT is often used. In empyema, a split pleura sign can be appreciated, showing thickening and separation of the visceral and parietal pleura. Increased pleural enhancement and edema of extrapleural fat are additional characteristics of empyema on CT [7].

## Conclusions

Although most lung abscesses are polymicrobial, the most common pathogens isolated in these infections include both aerobic and anaerobic *Streptococci*, *S. aureus*, and several gram-negative organisms. We present this case of a 51-year-old woman with an intraparenchymal lung abscess with the presence of *Peptostreptococcus *and *Veillonella *species. Our patient did not have a significant medical history of conditions that could have increased her risk of aspiration. She denied drinking alcohol or ever being diagnosed with neurological or esophageal disease. She does have a significant smoking history, however, she denies ever being diagnosed with pulmonary disease. The underlying cause of her lung abscess remains unknown, and the microbiological findings from this region were unforeseen. We strive to elucidate the distinctive clinical presentation evident in this case, alongside a comprehensive understanding of the unusual pathogens identified in the disease's pathogenesis.
